# Tracing Knowledge Gaps: Investigating the Influence of Education on News Exposure and Knowledge Using Digital Trace Data

**DOI:** 10.1177/19401612251335372

**Published:** 2025-05-18

**Authors:** Dominique S. Wirz, Ernesto de León, Silke Adam, Mykola Makhortykh

**Affiliations:** 1 Amsterdam School of Communication Research (ASCoR), University of Amsterdam, Amsterdam, Switzerland; 2 Institute of Communication and Media Studies, University of Bern, Bern, Switzerland

**Keywords:** knowledge gap, media use, COVID-19, education, digital trace data, web tracking, linkage analysis

## Abstract

The knowledge gap hypothesis—the assumption that an increasing flow of news on a topic fosters a gap in knowledge between the more and the less educated—has been demonstrated in numerous studies throughout the past 60 years. Knowledge gaps are attributed to individual differences in media selection and information processing capacities. However, it has been difficult to investigate the relative influence of selection and processing with conventional research methods. We used an innovative combination of individual-level digital trace and survey data collected in Switzerland (*n* = 403) and Germany (*n* = 471) to study the widening of knowledge gaps throughout the communication process. The data were collected at the onset of the COVID-19 pandemic, an extraordinary period of extremely high information inflow on a novel topic. Our analyses show that individuals with lower education use less online news in general and less COVID-19-related news in particular than those with higher education, which results in a difference in knowledge about the origin of COVID-19 (but not on its severity). However, those with lower education do not have a similar share of COVID-19-related news in their news diet, and they learn even more than those with higher education from the COVID-19-related news that they are exposed to. Our study thus suggests that knowledge gaps are predominantly a result of selecting into news use.

The knowledge gap hypothesis, first proposed by Tichenor et al. (1970), asserts that as mass media disseminate information, individuals with higher socio-economic status (SES) acquire this information more rapidly and effectively than those with lower SES. To date, numerous studies have supported the assumption that the media reinforce rather than reduce knowledge gaps between different segments of the population (for reviews, see Gaziano 1983; Hwang and Jeong 2009; Lind and Boomgaarden 2019; Viswanath and Finnegan 2012)—whereby SES has usually been operationalized with education. Knowledge gaps are particularly driven by print and online media and manifest for topics that get a lot of media coverage but that are not highly contested (Lind and Boomgaarden 2019). Further, they are strongest for factual knowledge, weaker for beliefs, and do not exist for issue awareness (Hwang and Jeong 2009).

Despite the large number of studies investigating the knowledge gap hypothesis, relatively little is known about the underlying mechanisms of the effect throughout the communication process. Bonfadelli (2002) proposed three stages in which gaps may arise. First, individuals with different levels of education may use different types of media and thus have *access* to different types of information. For example, individuals with higher education are more likely to visit a news website. Second, even if people access the same medium, they may *use* different types of content. For example, on a news website, individuals with higher education are more likely to select articles about hard news topics. And third, even if people use the same content, they may *process* the information differently. For example, individuals with higher education may find the news content easier to understand because they have more prior knowledge about a topic. Knowledge gaps can thus be fostered by differences in access to, use of, and processing of information (see also Lind 2020). However, to date, we do not know how influential each of these stages is for the widening of knowledge gaps.

To investigate to what extent individuals with different education levels differ in their access to news, use of topical information, and processing of this information, we use digital trace data and survey data collected from two samples: adults in Switzerland (*n* = 403) and in Germany (*n* = 471). The data were collected during the first months of the COVID-19 pandemic (March 17 to May 25); this context provides excellent conditions to study how knowledge gaps widen throughout the communication process. There was an extraordinarily high amount of news coverage on a novel topic; thus, knowledge gaps were likely to manifest. Studies confirm that in the early stage of the pandemic, individuals with higher education had higher knowledge about COVID-19 than those with lower education (Gerosa et al. 2021; Melki 2022; Wang et al. 2021). At the same time, it was literally vital that all parts of the population would be well informed about the threat posed by the virus and about preventive measures; therefore, knowledge gaps were extremely undesirable. Hence, it is highly relevant to investigate at which stage of the communication process, these gaps widened in order to learn what could prevent this in critical situations in the future.

Our data allow us to analyze to what extent individuals with low, medium, and high education differ in their access to news websites, their use of COVID-19-related news on such websites, and their knowledge about COVID-19. The digital trace data show that individuals with lower education used less online news and specifically less COVID-19-related news than those with higher education. In line with this, the survey data show a difference in knowledge about the origin of COVID-19 (but not on its severity) between those with lower and higher education. However, within their news diets, individuals with lower education did not select a significantly smaller share of COVID-19-related articles than those with higher education. Further, the correlation between the use of COVID-19-related news and knowledge was more positive for individuals with low education than for those with high education. Our findings thus suggest that knowledge gaps are predominantly driven by the access to news, less by the selection of relevant news topics, and *not* by different capabilities to learn from media content.

## The Knowledge Gap Hypothesis and Its Mechanisms

In their seminal work on the knowledge gap hypothesis, Tichenor et al. (1970) have discussed several factors that foster knowledge gaps. They mentioned *communication skills* (the ability to understand information provided by mass media); *prior knowledge* (which helps to integrate new information); *selective exposure, acceptance, and retention* (based on interest and prior knowledge); *relevant social contacts* (that provide information); and the *nature of the media system* (hard news topics are usually covered in print media, which are predominantly used by higher educated people). These factors can influence media selection and media reception (Bonfadelli 2002; Lind 2020); the media system and social contacts influence access to news, selective exposure influences the selection of news items within the accessed medium, and prior knowledge, communication skills, and selective acceptance and retention influence learning from the selected news items.

The digital information environment seems to amplify these processes. According to a meta-analysis, the internet is the medium that most strongly drives knowledge gaps (Lind and Boomgaarden 2019). Information on the internet is abundant and much more heterogeneous than in traditional mass media; the impact of access and use of information thus exerts a stronger influence on knowledge gaps than with traditional media (Bonfadelli 2002; Wei and Hindman 2011). Moreover, journalistic gate-keeping in traditional media does not only reduce the amount of information that media users may select, but editing practices—such as the placement or the size of a newspaper article—can also serve as cues for the relevance of a message and thus foster attention and thorough processing of information (Yang and Grabe 2011). On the internet, where journalistic editing is less prevalent and visible, media selection and information processing are highly dependent on users’ individual skills (Hargittai and Micheli 2019). This exacerbates knowledge inequalities.

Next to characteristics of the media (environment), also the nature of topics can influence the access, use, and processing of media content. For highly contested topics, such as climate change, knowledge gaps do not manifest (Lind and Boomgaarden 2019). It can be argued that such contested topics are relevant to all segments of the population, and thus, media users with low SES are equally motivated to seek information as those with higher SES (Kwak 1999). Further, such topics might be more frequently discussed outside of elite media and in interpersonal contexts. On the other hand, it may also be that the media report frequently about these topics but without adding new information. It is not the amount of media coverage but the amount of novel information that is circulated about a topic that fosters knowledge gaps (Wirth 2006). In general, it is assumed that individuals with higher education have developed better abilities to encode, store, and retrieve information; therefore, they can acquire new information more efficiently (Grabe et al. 2000, 2009). Individuals with lower education thus need more time (more repetition) to process the information to the same extent; when media coverage becomes redundant, individuals with lower SES can catch up on knowledge and the gap narrows (Wirth 2006).

## The Knowledge Gaps During the COVID-19 Pandemic

The COVID-19 pandemic provides a critical case for the study of knowledge gaps. From a public health perspective, it was extremely important that all segments of the population would understand the danger presented by the virus and know how to protect themselves and others. At the same time, knowledge gaps were very likely to arise: At the onset of the pandemic, people were confronted with a large and steady inflow of information on a topic that was novel for most. The topic was highly publicized, but not contested yet. In line with these theoretical considerations, studies from across the globe that were conducted in February to April 2020 have found that those with higher education had higher knowledge about COVID-19 (Gerosa et al. 2021; Melki 2022; Wang et al. 2021).

Even though these studies are all cross-sectional surveys, they can demonstrate the opening of a knowledge gap as all segments of the population had no prior knowledge about COVID-19 before it emerged. But how did media use contribute to the widening of this gap? Wang et al. (2021) found that internet use significantly increased COVID-19 knowledge in China, while Gerosa et al. (2021) found that media use, and especially social media use, had a negative impact on knowledge in the United States, and Melki (2022) found a gap narrowing effect for social media news exposure in Lebanon. These inconsistent findings highlight that the mere quantity of media use along different channels may not be a sufficient predictor of COVID-19 knowledge. As discussed before, knowledge gaps can be caused by individual differences in media selection—both in terms of access to media and use of specific media content is selected within those outlets—and information processing (Bonfadelli 2002; Lind 2020). Thus, access, use, and processing of information have to be considered to explain how individuals with different education levels learned about COVID-19.

## Methodological Challenges in Knowledge Gap Research

It is, however, very difficult to disentangle these effects with conventional research methods. Most survey-based approaches have measured general media exposure to a channel (e.g., newspapers) and attention to topical information within this channel (e.g., Cacciatore et al. 2014). Some researchers have considered that this media use measure is confounded with interest in the topics under study, as it is already the result of a selection process and have therefore statistically controlled for this (Fraile and Iyengar 2014). Nevertheless, measures of self-reported media exposure have at best a moderate reliability (Scharkow 2019) as they are based on participants’ ability to accurately recall and report their media use. Further, even when controlling for topic interest, these survey-based approaches cannot differentiate between effects of media selection and information processing.

Other researchers have partially manipulated media exposure by giving some participants access to a news website, while others did a different task and then observing which content of the news website was used and how this related to knowledge about the topics (Eveland and Schmitt 2015). This method enables testing of how access and content selection affect knowledge; however, it generates an artificial situation where access to the site is forced. Similarly, Bélanger et al. (2022) provided a sample of corporate trainees with a daily newspaper for a period of 10 months and afterward found that their knowledge on privacy and security topics had increased more than the knowledge of their peers. Even though this field study allowed for more freedom in media use and selective exposure to content within the newspaper, access was forced. Finally, researchers also experimentally tested the effects of exposure to TV, print, and online news and found that individuals with lower education learned more from TV news, while those with higher education learned more from newspapers (print and online). This approach isolates the effect of information processing, but individuals might be much more motivated to process information that they self-selected compared to such a forced exposure.

**Figure 1.**
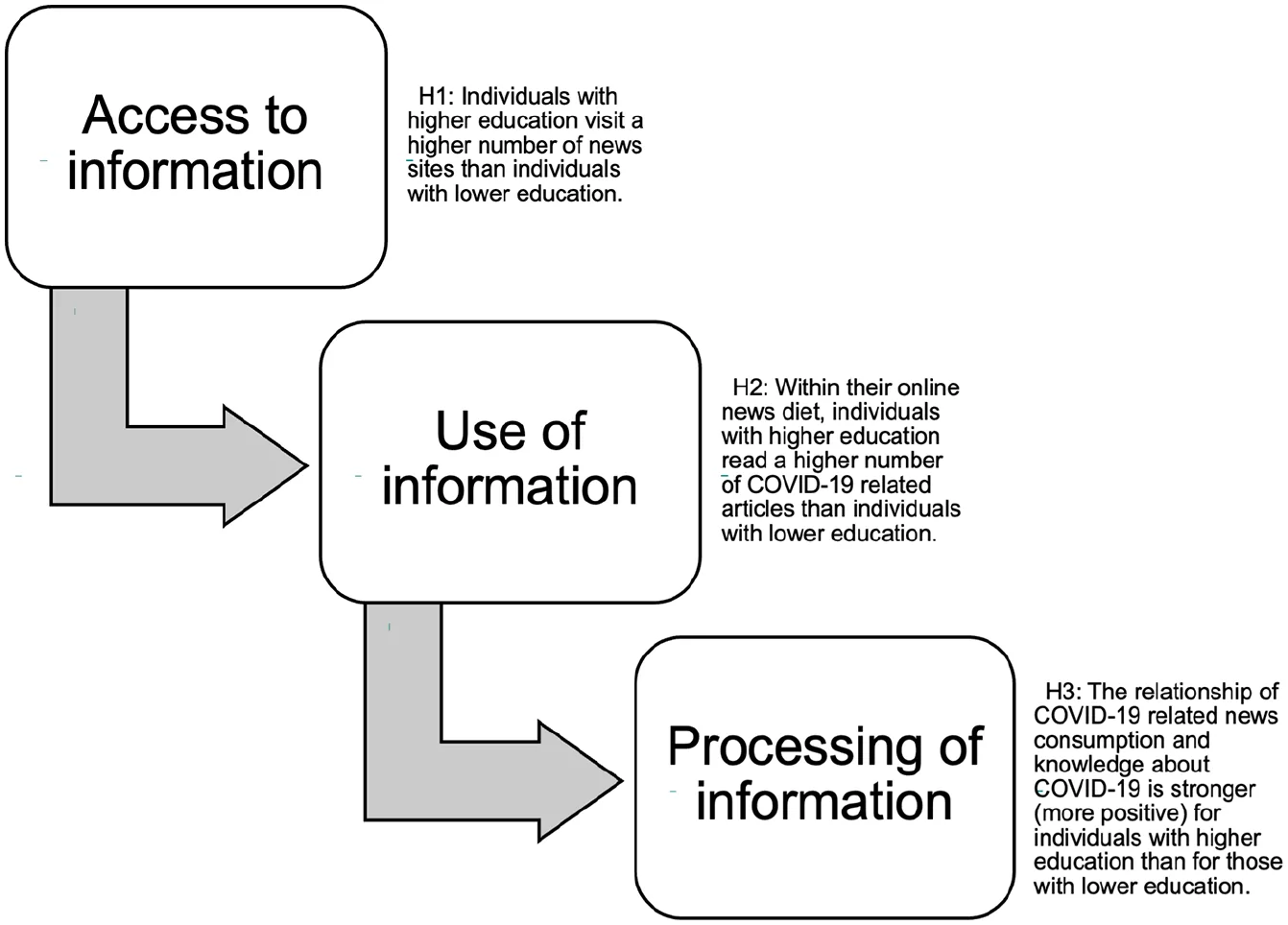
Formation of knowledge gaps throughout the communication process.

In summary, previous research has demonstrated that knowledge gaps can be a result of access, use, and processing of information (see Figure 1 for a visualization). However, it remains unclear which step of the communication process is more influential. Survey-based approaches can only detect the combined effect of access/use and processing, while experiments create artificial situations where access or use of information is forced, which can affect the motivation to process information (either in a positive way, in the sense of a demand characteristic, or in a negative way, in the sense of motivated attention and processing). To investigate how education affects each step of the communication process that can potentially foster knowledge gaps, we use a combination of digital trace data (obtained through online web tracking) and survey data collected at the onset of the COVID-19 pandemic. This approach allows us to see to what extent, individuals accessed online news sites, used COVID-19-related news content on these sites, and how exposure to COVID-19-related news content is related to knowledge about the issue.

Based on the knowledge gap hypothesis and considerations about how it develops through access, use, and processing of information processing (Bonfadelli 2002; Tichenor et al. 1970), we propose the following hypotheses:

H1: Individuals with higher education visit a higher number of news sites than individuals with lower education.
H2: Within their online news diet, individuals with higher education read a higher number of COVID-19-related articles than individuals with lower education.
H3: The relationship of COVID-19-related news consumption and knowledge about COVID-19 is stronger (more positive) for individuals with higher education than for those with lower education.

## Method

This study took advantage of an existing data set (Adam et al. 2024; Maier et al. 2024) that was inadvertently collected during an extraordinary period of extremely high information inflow on a novel topic.

### Sample

This research incorporates a blend of surveys and passively gathered web behavior data. Two quota-based samples were sourced from prominent online panels in Germany (DE) and the German-speaking region of Switzerland (CH) (Dynata, GapFish, demoSCOPE). Web tracking was conducted from March 17 to May 25, 2020, followed by a survey carried out between May 15 and May 25, 2020. Only participants who completed this survey and the web tracking (see “Web Tracking Data” section) were considered for the study, resulting in a total sample of 471 participants in Germany and 403 in Switzerland. Given the timing of the data collection, which coincided with the onset of the pandemic, the setup is ideal for studying how the public gathers information about a new topic and potentially leads to disparities in knowledge acquisition.

### Digital Trace Data Collected via Web Tracking

To precisely measure media consumption (Prior 2013), participants were asked to install a custom-developed plugin on their desktop browsers. This plugin passively monitored their browsing activities, capturing not only the URLs they visited but also scraping the HTML content of these pages. This method allowed for comprehensive data collection, surpassing many commercial solutions that often track only a limited set of websites or capture only URL or domain level data, but not the actual content. By capturing both the URLs and the actual content accessed, this approach addresses gaps identified in recent studies (see Gonzalez-Bailon & Xenos 2023) and facilitates automated content analysis to identify new areas of interest. The web tracking data were combined with a survey on demographics and knowledge, enabling the analysis of individual opinions and attitudes alongside actual media consumption behavior. The benefits of this innovative approach have been extensively discussed in recent literature (Christner et al. 2022; Stier et al. 2020).

Participants provided informed consent for tracking, with the conditions clearly outlined. To protect sensitive information, the tracker employed a “denylist” system, excluding domains related to sensitive topics (e.g., banking, insurance, email services, explicit content). Participants also had the option to pause the tracker for 15-minute intervals at any time. Although some participants opted out of tracking, which might correlate with certain variables of interest and introduce potential bias, prior research (Gil-López et al. 2023) found minimal differences in political variables (e.g., political interest, trust, and participation) between those who participated and those who did not. An analysis of the demographic composition of our sample relative to the populations of Switzerland and Germany showed that our web tracking samples generally reflected the age distributions of both countries, with a slight over-representation of older participants (average age of 44 in Switzerland and 49 in Germany, compared to 42 and 45 in the general population, respectively). However, there was an under-representation of less-educated individuals (DE: 13% vs. 12% population quota; CH: 3% vs. 14% population quota) and an over-representation of more-educated individuals (DE: 36% vs. 26.8%; CH: 41% vs. 37%). Male participants were also over-represented (DE: 56% vs. 49.7%; CH: 56% vs. 49.3%).

Participants who registered fewer than 5 days of online activity were excluded from the analysis to avoid cases where the tracker was installed but not used. Despite the method’s strengths and the precautions taken, one significant limitation remains: the web tracker only captures content viewed on desktop browsers. With the increasing reliance on mobile devices for information consumption, this limitation must be considered when interpreting the study’s results. Therefore, web tracking should be viewed as a tool that enhances our understanding of online information behavior but does not capture it entirely. The data we collect should be seen as a sample of our online behavior, providing a good approximation of our habits while recognizing its incomplete nature.

The web tracking plugin developed for this project has since been converted into an open-source tool maintained by GESIS—Leibniz Institute for the Social Sciences and is available for public use.^1^

### Instrument

#### Web Tracking Measures

We constructed three exposure measures. First is exposure to news in general. This was done by matching participants’ web tracking data to a list of established German and Swiss news sources taken from previous work (de León et al. 2023). On average, participants in the sample visited a news site 153.1 times, the least visits is 0, and the most visits by a participant is 2,577 visits. Second, we constructed a measure of exposure to COVID-19 news by counting the number of news articles accessed that mention “covid19” OR “coronavirus” OR “COVID-19” at least three times (Min = 0, Max = 1,398, Mean = 69.6), as past work has shown this to be a good threshold that minimizes the rate of false positives (de León et al. 2023). We operationalized COVID-19 news exposure in two ways: both as total count of news stories on COVID-19 visited, and second as the share of COVID-19 news to all news consumed (as this accounts for general news readership).

Finally, we constructed a measure capturing how many times participants read news on the origin of COVID-19 (at the time, the Wuhan market theory, where the virus was passed from wildlife to humans, was most prominent). To do this, we registered the times participants visited news on COVID-19 that also included at least three mentions of “fledermause”/“fledermäuse” (bat in German) OR “Wildtiere”/“Wildtieren” (wildlife in German) (Min = 0, Max = 105, Mean = 3.1).

#### Knowledge Measures

**Figure 2.**
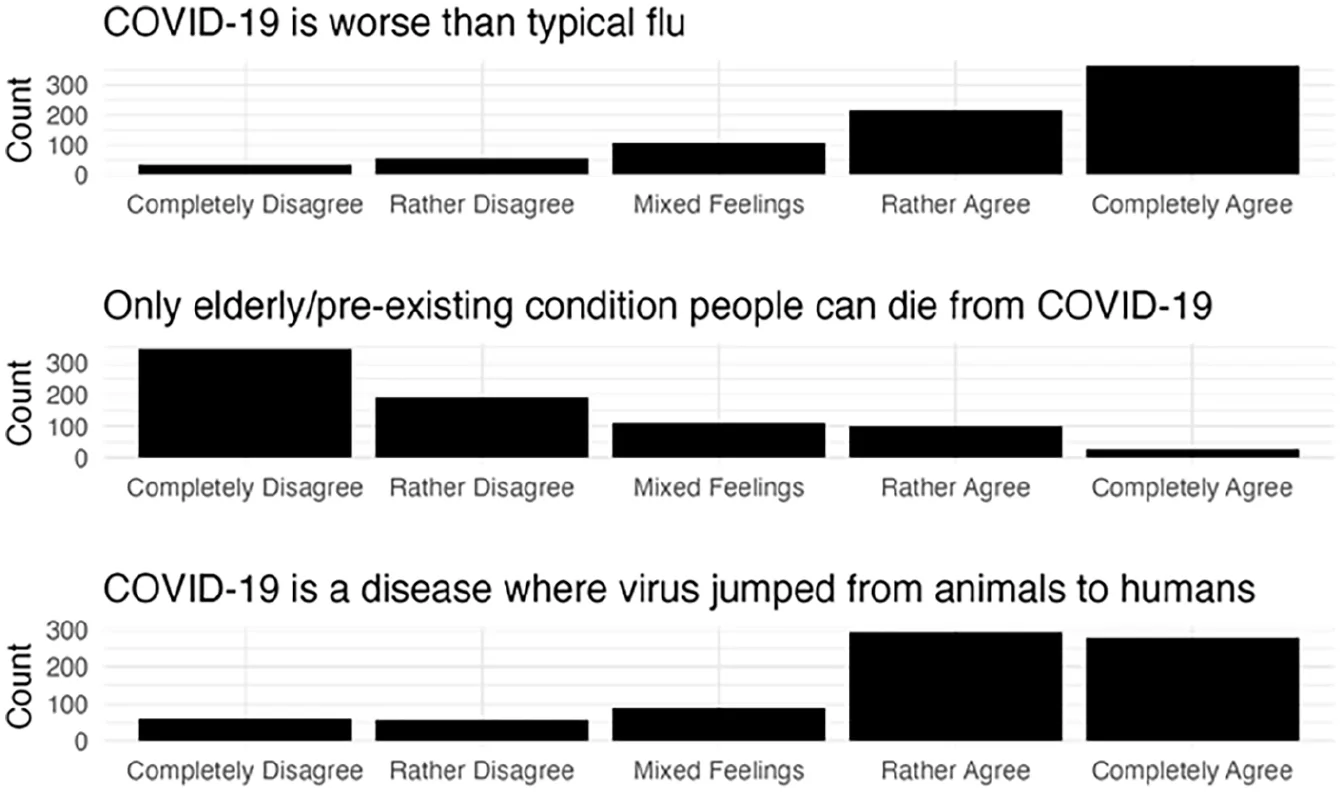
Distribution of answers to knowledge questions.

To construct the knowledge measure, three variables were used to reflect the accuracy of participants’ knowledge of the COVID-19 pandemic. Participants had to register their agreement or disagreement with the following statements: “COVID-19 ist schlimmer als eine typische Grippe” (“COVID-19 is worse than the typical flu”), “Nur alte Menschen und Menschen mit Vorerkrankung können an COVID-19 sterben” (“Only elderly people and those with pre-existing conditions can die from COVID-19”), and “Corona ist eine Krankheit, bei der ein Virus vom Tier auf den Menschen übergesprungen ist” (“Corona is a disease where a virus jumped from an animal to a human”). Answers, originally given on a five-point Likert scale, were recoded into binary indicators of correctness (see Figure 2 for the distribution on the original scale). For the first and last items, responses indicating a high level of agreement (4 and 5) with statements deemed factually incorrect were coded as correct, while those reflecting disagreement (1, 2, and 3) were coded as incorrect. Conversely, for “only elderly people and those with pre-existing conditions can die from COVID-19,” responses disagreeing with the statement were coded as correct, and those that did not were coded as incorrect. These recoded variables were then summed to create a composite knowledge score, ranging from 0 to 3, where higher values indicate a greater number of correct responses. Notably, the project was not conceptualized to measure knowledge about COVID-19; it was a coincidence that the field time started at the onset of the pandemic. Therefore, the knowledge measure is not as comprehensive as in typical studies, but the items clearly represent factual knowledge about COVID-19 that was adequate and relevant at the time of the study.

#### Education

Education levels were measured based on the logic of the International Standard Classification of Education (ISCED, see Appendix 2). In Switzerland, this was done across twelve categories, ranging from “Obligatory schooling not completed” to “University level education.” In Germany, it included eleven categories, from “No qualification” to “University level education.” Because these categories are not ordinal and are hard to compare against each other, we followed the ISCED logic in forming three educational groups: the low education group contains people who stayed below secondary education (7.32% of our sample), the middle education group consists of those with secondary education (52.6% of our sample), whereas the high education group contains those with tertiary education (40% of our sample).

#### Control Variables

In the analysis, we also make use of three key demographic variables: age, gender, and country. The participants’ ages ranged from 18 to 75 years (*M* = 47.31), and men are overrepresented (56.64%) and are more Swiss (53.89%) than German (46.11%).

### Design and Analysis

We conducted a linkage analysis combining the longitudinal web tracking data (news exposure) to the cross-sectional survey data (education, knowledge). To evaluate the relationship between education and news consumption (H1 and H2), we modeled news consumption as a function of education, while controlling for age, gender, and country. We build on previous work estimating the effect of individual-level characteristics on website visits (e.g., de León et al. 2024), using a negative binomial regression model to account for the zero-inflated distribution of the variable capturing news readership and COVID-19 news readership. Negative binomial regression models are particularly suited for this analysis because they account for overdispersion in count data, where the variance exceeds the mean. In the context of news readership and COVID-19 news readership, many individuals may consume little to no news, leading to an excess of zero counts that a standard Poisson model would struggle to accommodate. By incorporating a dispersion parameter, the negative binomial model provides more accurate standard errors and reliable inferences, ensuring a better fit for the observed data distribution (Ver Hoef and Boveng 2007). To evaluate the effect of news readership and education on knowledge (H3) an ordinary least squares (OLS) regression model was used, which modeled the composite knowledge variable as a result of education and news consumption while controlling for age, gender, and country. Additionally, an exploratory analysis was conducted (see results). For these, OLS regression models were employed. We provide standardized versions of all regression analyses conducted in Appendix Tables A2–A4.

Replication materials for all analyses can be found in the following repository: https://osf.io/a2k3v.

## Results

**Figure 3.**
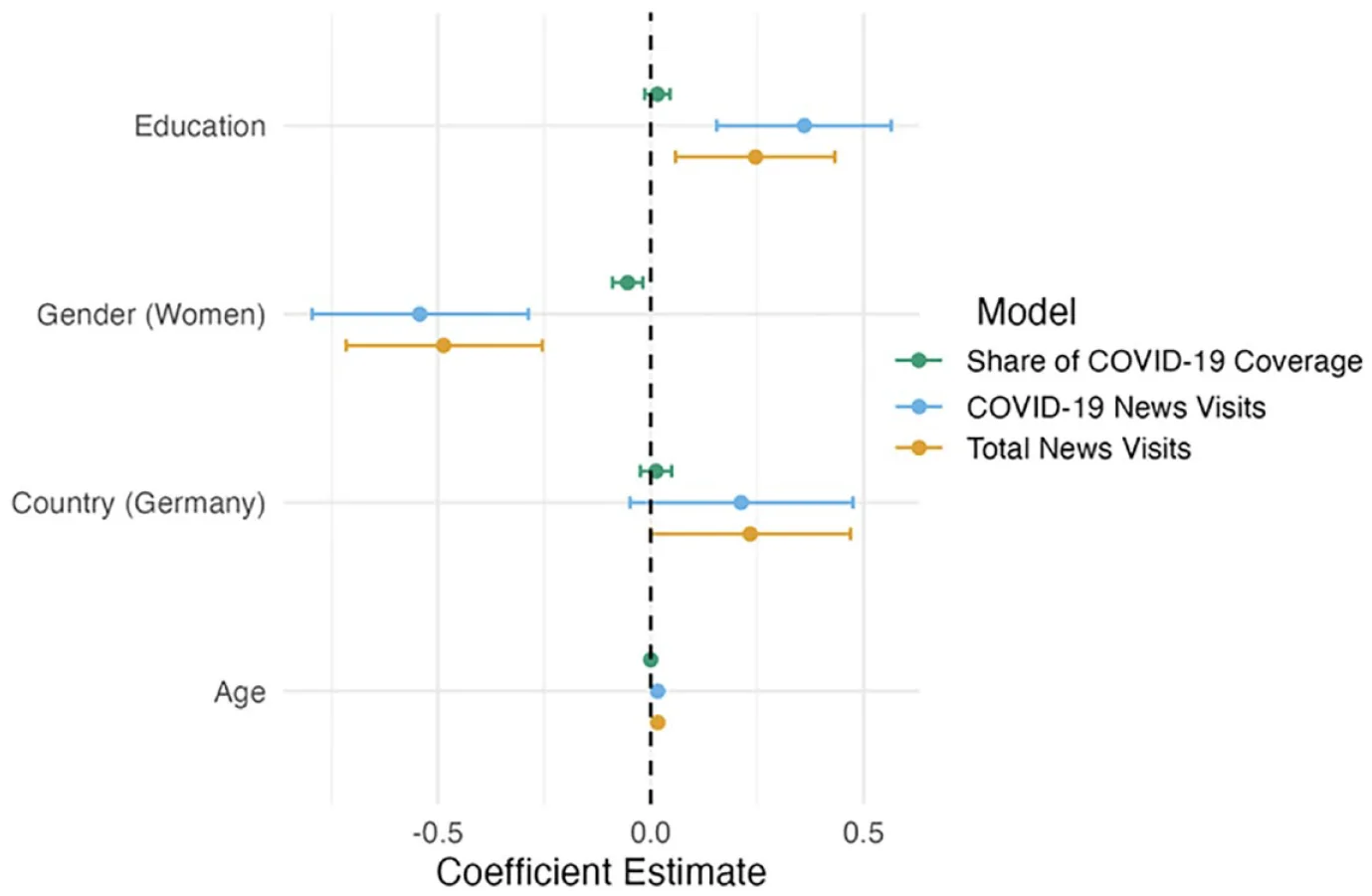
Negative binomial models with news consumption as a function of education.

Figure 3 allows us to address the relationship between education and news consumption. It plots the results of two negative binomial regression models where total news consumption (model 1) and COVID-19 news consumption (model 2) are modeled as the result of education, while controlling for gender, country of origin, and age. In the first model, there is a positive effect of education on total news consumption (0.246), which is statistically significant (*p* < .01). This finding confirms H1, indicating that higher education is associated with increased overall news consumption. In the second model, education has an even stronger positive effect on COVID-19 news consumption (0.361), which is highly statistically significant at the *p* < .001 level. This provides initial support for H2, suggesting that individuals with higher education levels consumed more COVID-19 news compared to those with lower education levels. Finally, the third model estimates the effect of education on the relative share of COVID-19 in participants’ news diets. Here, we observe a positive but statistically insignificant effect. This does not support H2—it appears that those with higher education consume more general news, more news on COVID-19, but not a greater share of COVID-19 relative to overall news consumption. To more carefully examine this relationship, Table 1 displays the mean and median general news, COVID-19, and share of COVID-19 news for each education group. We can observe that, overall, the share of COVID-19 news does increase with education level, but this relationship disappears when controlling for gender, country, and age. Thus, overall, we find mixed evidence for H2: Higher exposure to COVID-19 news among those with higher education seems to be mostly a result of higher overall news use, but not a result of the selection of specific content within the news diet.

**Table 1.** News Consumption by Education Levels.

Education level	Total news (mean)	Total news (median)	COVID-19 news (mean)	COVID-19 news (median)	Share of COVID-19 news (mean)	Share of COVID-19 news (median)
1	151,734	27.5	62,953	9.5	0.388	0.397
2	130,696	29	55,567	8.5	0.405	0.413
3	182,766	35	89,303	12	0.418	0.429

For all models in Figure 3, gender has a negative effect on news consumption (*p* < .001), implying that women consume less news than men, while age has a positive effect, indicating that older individuals tend to consume more news.

**Figure 4.**
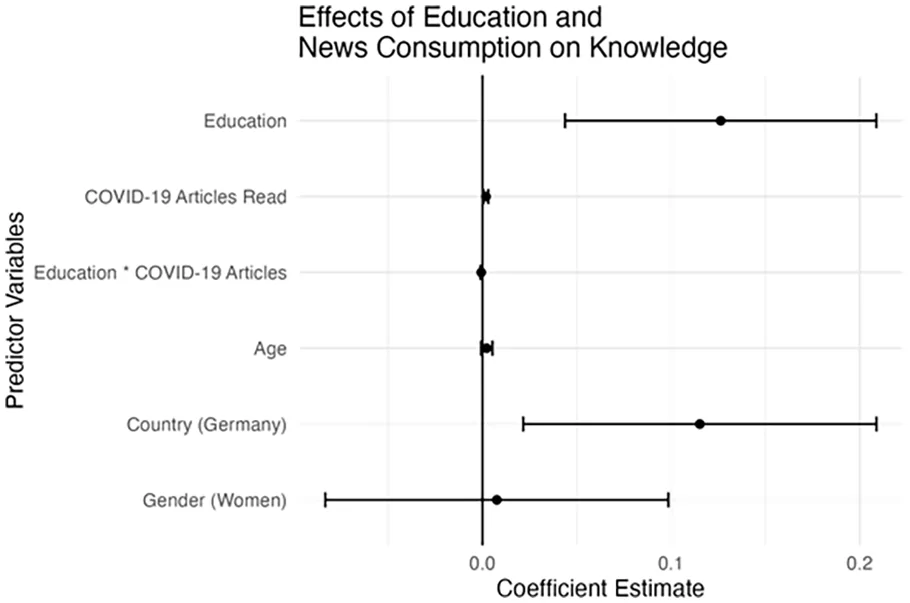
Result of OLS model predicting COVID-19 knowledge as a function of education and news consumption. *Note.* OLS = ordinary least squares.

Figure 4 shows an OLS regression model that estimates the relationship between knowledge about the COVID-19 pandemic, education, and news consumption. We first observe that education has a strong and positive effect on knowledge with a statistically significant (*p* < .01) unstandardized coefficient of 0.120, confirming the basic assumptions of the knowledge gap. While the effect of COVID-19 news visits is in the correct positive direction (β = 0.001), the effect is statistically insignificant. To evaluate H3, which states that the effect of COVID-19-related news consumption on knowledge will be stronger for those with higher education, the model includes an interaction effect between COVID-19 news visits and knowledge. Nevertheless, the effect is in the opposite direction (β = −0.0003) and statistically insignificant. We, therefore, reject H3.

### Exploratory Analysis

To investigate the (seemingly non-existent) relationship between education and learning from COVID-19-related news, we evaluate the relationship between education, news, and knowledge for each knowledge item individually, as previous research has shown that knowledge gaps differ depending on the type of knowledge measured (Hwang and Jeong 2009). We provide these results in Table 2. While models 1 and 2, which used questions about whether COVID-19 was worse than the common flu and the idea that only the old and ill can die of the virus as measures of knowledge, show similar non-results to the previous assessment, a different pattern emerges for the knowledge question related to the origin of COVID-19. We see a strong, positive effect (β = 0.350) of education on knowledge about the origin of the virus (*p* < .001). Similarly, we can observe a statistically significant (*p* < .05) positive relationship between the number of articles about COVID-19 articles visited and knowledge about the origins of COVID-19 (β = 0.003). Interestingly, the interaction term between visits of articles about COVID-19 read and education is in the negative direction—contrary to what was expected—and approaching significance (*p* < .1). This interaction term suggests that the relationship between COVID-19 news consumption and knowledge about the origin of the virus is stronger for those with lower education.

**Table 2.** Regression Models Predicting Individual COVID-19 Knowledge Items.

Predictors and model fit statistics	Dependent variable:		
	COVID-19 worse than flu	Only old and ill COVID-19	COVID-19 origin
	1	2	3
Education	−0.080 (0.071)	−0.054 (0.073)	0.350*** (0.078)
Age	−0.012*** (0.003)	0.010*** (0.003)	0.001 (0.003)
Gender (women)	0.008 (0.078)	0.141 (0.080)	−0.139 (0.086)
Country (Germany)	−0.060 (0.080)	0.410*** (0.082)	0.055 (0.088)
COVID-19 articles read	−0.001 (0.001)	0.001 (0.001)	0.003* (0.001)
Education × COVID-19 articles read	0.0002 (0.0004)	−0.001 (0.0004)	−0.001 (0.0005)
Constant	2.860*** (0.260)	2.810*** (0.266)	3.057*** (0.283)
Observations	861	868	789
*R* ^2^	0.039	0.062	0.042
Adjusted *R*^2^	0.032	0.055	0.034
Residual std. error	1.135 (df = 854)	1.167 (df = 861)	1.190 (df = 782)
*F* statistic	5.752*** (df = 6; 854)	9.413*** (df = 6; 861)	5.645*** (df = 6; 782)

* *p* < .05. ***p < .001.

Does this difference in knowledge about one aspect of COVID-19 result from differences in how often this information is encountered in a person’s media diet? To further investigate this, we construct a new variable that registers not only how many articles on COVID-19 users read, but the number of articles particularly providing the information that the COVID-19 virus originated from a wildlife market in Wuhan. To do this, we recorded the number of articles seen by participants that mention COVID-19 AND bats OR wildlife (see Methods section). We then estimated the effect of reading this specific type of information on knowledge about the origin of the virus. In Table 3, we see that the news consumption effect is much stronger: while in the previous model (Table 2), the unstandardized effect was of β = 0.003 (standardized β = 0.374), in Table 3, it is of β = 0.070 (*p* < .01) (standardized β = 0.516). Similarly, we see that the interaction effect is also stronger but still in the negative direction, with an unstandardized coefficient of β = −0.023 (standardized β = −0.449) and statistically significant (*p* < .05). This means that the effect of consuming news on the origins of the virus on knowledge of the origin of the virus is stronger for those with lower education.

**Table 3.** Regression Model Predicting COVID-19 Origin Item as a Function of COVID-19 Origin News Articles Consumption.

Predictors and model fit statistics	Dependent variable
	COVID-19 origin
Education	0.343*** (0.074)
Age	0.001 (0.003)
Gender (women)	−0.156 (0.085)
Country (Germany)	0.098 (0.089)
COVID-19 origin articles	0.070** (0.026)
Education × COVID-19 origin articles	−0.023* (0.010)
Constant	3.050*** (0.279)
Observations	789
*R* ^2^	0.042
Adjusted *R*^2^	0.035
Residual std. error	1.189 (df = 782)
*F* statistic	5.729*** (df = 6; 782)

* *p* < .05. **p < .01. ***p < .001.

Thus, those with higher education are more often confronted with information about the origin of the virus, which explains why they know more about this aspect than those with lower education. However, if individuals with lower education see information about the origin of COVID-19 in the media, they are even more likely to learn it than those with higher education. This seems counterintuitive but is presumably a threshold or ceiling effect: only a few individuals with lower education were exposed to information about the virus’ origin (18 participants in our sample); therefore, we see a clear positive relationship between exposure and knowledge for this group. Within the group of highly educated individuals, exposure was more frequent, and at some point, more exposure does not generate more knowledge, so the relationship between exposure and knowledge disappears. Overall, our findings thus suggest that those who were exposed to information about the origin of COVID-19 learned from it, and this was just as much the case for those with lower and with higher education. Hence, the differences in knowledge between socio-economic groups can be attributed to which information they encounter in the media, and not their ability to process the information.

## Discussion

Overall, our study—in line with others that have been investigating this (Gerosa et al. 2021; Melki 2022; Wang et al. 2021)—demonstrates, first, that knowledge gaps emerged during the COVID-19 pandemic; in our case, in Germany and Switzerland: the most educated had most knowledge about the pandemic. The knowledge gap, however, manifests solely regarding certain aspects of the topic: it is the origin of the virus where education plays a role, but not questions on its severity. Education thus matters more for background information. Yet, life-relevant practical information seems to reach individuals independent of their education. On the one hand, this is good news; the most important (lifesaving) information was adopted equally across educational groups. On the other hand, background information was not adopted equally. This may increase susceptibility for misinformation (see also Gerosa et al. 2021). Specifically, there were many conspiracy theories about the origin of the virus, and individuals who did not know what was consensus among scientists may have been more open to believe in alternative explanations. Our findings thus stress the challenge of reaching out to the less educated with information that does not directly affect their own life, but asks broader, societal, and political questions.

The second, and maybe more important, finding is that although individuals with higher education used more news (in general and about COVID-19), they did not have a significantly higher share of COVID-19-related news in their news diets, and they did not learn more from the news on COVID-19 that they were exposed to—on the contrary. Thus, it is the mere quantity of news use that predicted knowledge gaps. Considering the funnel that Bonfadelli (2002) has outlined, it thus seems that the critical factor is access, while selection within a news outlet and information processing do not play a major role. If these findings were to hold beyond the specifics of an outbreak of a pandemic, knowledge gap theory would boil down to a theory of education-related selection processes emphasizing the role of social economic status for selecting into news. This is an important contribution as most research on selection or avoidance of news about politics relies on political interest or content preferences (e.g., Prior 2005; Strömbäck et al. 2013) instead of revealing the underlying social structure, that is, investigating how SES may influence these individual-level predictors.

Third, our study indicates that the argument that individuals with higher education have the capacity to process information more thoroughly does not hold for all topics. At the outbreak of the pandemic, our findings show that the least educated profit most from exposure. This finding might be specific for COVID-19, but it is not unique and might thus still be generalizable: For example, a recent study from China demonstrates that those groups of the population who are least likely to access the internet are those who benefit most from the knowledge returns of its use (Cheng 2023). Similarly, a study from Spain also found that the relationship between education and knowledge was weaker among heavy newspaper users than among light newspaper users (Fraile 2011). A likely explanation for this is that there is a ceiling effect; at some point, exposure to more news does not provide more information and thus not contribute to further knowledge; therefore, effects of exposure on knowledge are mostly visible in groups where exposure is low. In other words: reading one more news article about COVID-19 may not provide new insights to those who are already well informed, but it can contribute to a significant knowledge increase for those who are not yet well informed. In general, many media effects can only be measured at a specific point in time, as they do not reflect linear trends over time, but have a certain onset after which a stabilization occurs (see, e.g., Baumgartner 2024). Applied to our study, that means a new information is new only the first time it is encountered, and as soon as it is learned, further exposure to the same information cannot lead to further learning. This indicates that the processing aspect of the knowledge gap theory should not necessarily be taken as a factor pushing knowledge gaps, but more as a factor weakening educational inequalities—at least as regards topics less complex in nature. Compared to experimental studies that found a relationship between education and information processing, individuals may have a better ability to process the news content they self-select. That is, in self-directed news use, they choose news content that has an adequate difficulty level for their preferences and capabilities, and therefore, they are able to learn the information it provides.

If access is key in countering educational and status inequalities, a functioning media system with free and wide access to high-quality information is crucial. Switzerland and Germany are two countries with strong public service media, but our findings still suggest that the quantity of news consumption made the difference. A media system alone thus cannot overcome such inequalities. Instead, it needs for installing a constant motivation to make use of information possibilities—a challenge for schools and educational institutions well before universities.

Beyond these insights, our study also makes an important methodological contribution. Knowledge gap research using traditional methods such as surveys or experiments was limited to investigating either selection or learning effects. The use of web tracking data allows us to investigate both processes at the same time. Further, our approach allows us to measure the effect of self-selected media exposure on knowledge, whereas previous research always involved some sort of forced access. Our findings about the relationship of exposure and knowledge diverge from previous work in that we do not find that those with higher education learn more from the media content they encounter. This divergent finding may be a result of selective versus forced exposure to information. We, therefore, suggest that researchers make use of web tracking data to further investigate how natural selection processes influence information processing.

Of course, the present study has some limitations: First of all, we use data that were not collected with the purpose to test these hypotheses. This means that the data include only few knowledge items, which do not reflect the full spectrum of knowledge one could have about COVID-19 at that time. Nevertheless, the data set offers a unique opportunity to gain a better understanding about the development of knowledge gaps through media use, and the knowledge items have a good face validity. Second, the online tracking data do not capture an individuals’ whole news consumption, and not even their whole online news use. However, online news consumption on the desktop computer can be a good proxy for general news consumption and the data that we were able to obtain this way are more informational than survey measures of news use would be in the context of our research. It remains, however, open if desktop internet use is equally representative for general internet use across different levels of education. Further, it has to be noted that we only measured the quantity of exposure to (COVID-19-related) news but have no information on the quality (information-richness or accuracy) of these news.

Another potential limitation is that we only measured knowledge at one time point. While it can easily be argued that knowledge was absent before the start of the web tracking period, it would be important to investigate how knowledge developed further during the pandemic. Especially in the case of COVID-19, it is important to consider the necessity to update one’s knowledge as new information becomes available; this flexibility might also be affected by education. Beyond that, we do not know if individuals with lower education were able to catch up on knowledge later. Such idea is crucial as a study from Singapore conducted in a late stage of the H1N1 influenza pandemic shows that knowledge gaps between citizens with different education levels still existed, but that TV and interpersonal communication had a narrowing effect, while no type of media use was found to have a widening effect (Ho 2012). Thus, individuals with lower education may catch up when the information inflow is not high anymore, but the fact that knowledge gaps still existed several months after the pandemic’s peak demonstrates that inequalities persist.

Moreover, participants with lower education were underrepresented in our samples. This is quite usual in survey research, and especially in studies that require the installation of software on participants’ devices. However, it is an important limitation given the central role of education in our study. It might be that individuals with lower education in our sample are different from their peers who did not participate, for example, they may be more active on the internet. Nevertheless, we see that they use less news than those with higher education and have less knowledge about COVID-19. While this fosters confidence in the validity of our results, it would still be important to replicate these findings with a more balanced sample.

Finally, we must account for the exceptional circumstances during the COVID-19 pandemic. Many people had more time to consume media during the lockdown, the motivation to seek information on the topic was high, and journalists may have put more effort to convey information in an accessible manner. The fact that we do still find knowledge gaps, however, demonstrates that even in such exceptional circumstances, inequalities persist.

Despite these limitations, this study makes an important contribution to the study of knowledge gaps. Relying on web tracking data, we can show that the amount of COVID-19-related news exposure—but not relative share of COVID-related news in a person’s news repertoire—fosters knowledge gaps. Further, we find that those with lower education learned most in relation to the amount of news they consumed, but still had less background knowledge because of the overall lower news consumption. Thus, even for an issue as obtrusive as COVID-19, we find differences in knowledge acquisition between individuals with lower and higher education. These are mainly caused by differences in access to news, less by selection into news topics, and not by information processing.

## Appendix 1

### Regression Model Specifications

**Table A1.** Full Specifications for Models Displayed in Figures 3 and 4.

Predictors and model fit statistics	Dependent variable			
	Total news visits	COVID-19 news visits	Share of COVID-19 news	Knowledge
	1	2	3	4
Regression models				
Education	0.246* (0.096)	0.361*** (0.107)	0.016 (0.015)	0.120** (0.041)
Age	0.017*** (0.004)	0.016*** (0.004)	−0.0002 (0.001)	0.002 (0.002)
Gender (women)	−0.487*** (0.116)	−0.543*** (0.128)	−0.054** (0.018)	0.023 (0.045)
Country (Germany)	0.234 (0.120)	0.212 (0.133)	0.013 (0.019)	0.125** (0.047)
COVID-19 articles read				0.001 (0.001)
Education × COVID-19 articles read				−0.0003 (0.0002)
Constant	3.941*** (0.376)	3.007*** (0.417)	0.441*** (0.059)	0.879*** (0.150)
Observations	874	874	780	874
*R*^2^			0.013	0.022
Adjusted *R*^2^			0.008	0.016
Log likelihood	−4,770.599	−3,934.328		
Theta	0.345*** (0.014)	0.281*** (0.012)		
Akaike inf. crit.	9,551.197	7,878.657		
Residual std. error			0.252 (df = 775)	0.663 (df = 867)
*F* statistic			2.570* (df = 4; 775)	3.314** (df = 6; 867)

* *p* < .05. ***p* < .01. ****p* < .001.

**Table A2.** Table with Standardized Coefficients for Models Displayed in Figures 3 and 4 and Appendix Table A1.

Predictors and model fit statistics	Dependent variable			
	Total news visits	COVID-19 news visits	Share of COVID-19 news	Knowledge
	1	2	3	4
Regression models with standardized coefficients				
Education	0.0005* (0.122)	0.001*** (0.132)	0.037 (0.015)	0.109** (0.041)
Age	0.001*** (0.005)	0.002*** (0.005)	−0.013 (0.001)	0.040 (0.002)
Gender (women)	−0.001*** (0.148)	−0.002*** (0.158)	−0.107** (0.018)	0.017 (0.045)
Country (Germany)	0.0004 (0.153)	0.001 (0.164)	0.025 (0.019)	0.093** (0.047)
COVID-19 articles read				0.210 (0.001)
Education × COVID-19 articles read				−0.186 (0.0002)
Constant	(0.479)	(0.515)	(0.059)	(0.150)
Observations	874	874	780	874
*R*^2^	0.999	0.995	0.013	0.022
Adjusted *R*^2^	0.999	0.995	0.008	0.016
Log likelihood	−4,770.599	−3,934.328		
Theta	0.345*** (0.014)	0.281*** (0.012)		
Residual std. error	1.274 (df = 869)	1.235 (df = 869)	0.252 (df = 775)	0.663 (df = 867)
*F* statistic	192,030.100*** (df = 4; 869)	42,292.970*** (df = 4; 869)	2.570* (df = 4; 775)	3.314** (df = 6; 867)

* *p* < .05. ***p* < .01. ****p* < .001.

**Table A3.** Table with Standardized Coefficients for Table 2.

Predictors and model fit statistics	Dependent variable		
	COVID-19 worse than flu	Only old and ill COVID-19	COVID-19 origin
	1	2	3
Education	−0.042 (0.071)	−0.027 (0.073)	0.175*** (0.078)
Age	−0.164*** (0.003)	0.121*** (0.003)	0.014 (0.003)
Gender (women)	0.004 (0.078)	0.059 (0.080)	−0.058 (0.086)
Country (Germany)	−0.026 (0.080)	0.170*** (0.082)	0.022 (0.088)
COVID-19 articles read	−0.147 (0.001)	0.174 (0.001)	0.374* (0.001)
Education × COVID-19 articles read	0.083 (0.0004)	−0.176 (0.0004)	−0.298 (0.0005)
Constant	(0.260)	(0.266)	(0.283)
Observations	861	868	789
*R* ^2^	0.039	0.062	0.042
Adjusted *R*^2^	0.032	0.055	0.034
Residual std. error	1.135 (df = 854)	1.167 (df = 861)	1.190 (df = 782)
*F* statistic	5.752*** (df = 6; 854)	9.413*** (df = 6; 861)	5.645*** (df = 6; 782)

* *p* < .05. ****p* < .001.

**Table A4.** Table with Standardized Coefficients for Table 3.

Predictors and model fit statistics	Dependent variable
	COVID-19 origin
Education	0.172*** (0.074)
Age	0.012 (0.003)
Gender (women)	−0.065 (0.085)
Country (Germany)	0.040 (0.089)
COVID-19 origin articles	0.516** (0.026)
Education × COVID-19 origin articles	−0.449* (0.010)
Constant	(0.279)
Observations	789
*R* ^2^	0.042
Adjusted *R*^2^	0.035
Residual std. error	1.189 (df = 782)
*F* statistic	5.729*** (df = 6; 782)

* *p* < .05. ***p* < .01. ****p* < .001.

**Table A5.** Analyses Split by Country for Appendix Table A1 (Models 1, 2, 3, and 4).

Predictors and model fit statistics	Dependent variable							
	Total news visits		COVID-19 news visits		Share of COVID-19 news		Knowledge	
	Switzerland	Germany	Switzerland	Germany	Switzerland	Germany	Switzerland	Germany
	1	2	3	4	5	6	7	8
Education	0.397** (0.149)	0.138 (0.124)	0.552** (0.168)	0.205 (0.135)	0.027 (0.024)	0.010 (0.019)	0.119 (0.062)	0.126* (0.055)
Age	0.021*** (0.005)	0.009 (0.007)	0.020*** (0.006)	0.008 (0.007)	−0.001 (0.001)	0.0003 (0.001)	0.0003 (0.002)	0.005 (0.003)
Gender (women)	−0.261 (0.164)	−0.698*** (0.162)	−0.386* (0.184)	−0.709*** (0.177)	−0.030 (0.026)	−0.081** (0.025)	0.060 (0.063)	−0.017 (0.065)
COVID-19 articles read							0.001 (0.001)	0.001 (0.001)
Education × COVID-19 articles read							−0.0002 (0.0004)	−0.0003 (0.0003)
Constant	3.282*** (0.484)	5.320*** (0.513)	2.377*** (0.545)	4.420*** (0.560)	0.406*** (0.078)	0.491*** (0.078)	1.018*** (0.193)	1.012*** (0.210)
Observations	471	403	471	403	411	369	471	403
*R* ^2^					0.007	0.030	0.010	0.026
Adjusted *R*^2^					−0.001	0.022	−0.0005	0.014
Log likelihood	−2,467.462	−2,299.071	−2,014.986	−1,914.469				
Theta	0.322*** (0.019)	0.380*** (0.023)	0.254*** (0.016)	0.320*** (0.020)				
Akaike inf. crit.	4,942.923	4,606.143	4,037.972	3,836.939				
Residual std. error					0.266 (df = 407)	0.235 (df = 365)	0.678 (df = 465)	0.646 (df = 397)
*F* statistic					0.905 (df = 3; 407)	3.749* (df = 3; 365)	0.958 (df = 5; 465)	2.146 (df = 5; 397)

* *p* < .05. ***p* < .01. ****p* < .001.

**Table A6.** Analyses Split by Country for Table 2.

Predictors and model fit statistics	Dependent variable					
	COVID-19 worse than flu	Only old and ill COVID-19	COVID-19 origin		Switzerland	Germany
	Switzerland	Germany	Switzerland	Germany		
	1	2	3	4	5	6
Education	−0.089 (0.103)	−0.067 (0.099)	−0.077 (0.112)	−0.041 (0.094)	0.350** (0.116)	0.369*** (0.106)
Age	−0.011*** (0.003)	−0.016*** (0.005)	0.008* (0.003)	0.014** (0.004)	−0.002 (0.004)	0.007 (0.005)
Gender (women)	−0.045 (0.105)	0.061 (0.117)	0.259* (0.114)	0.012 (0.112)	−0.197 (0.118)	−0.075 (0.125)
COVID-19 articles read	−0.001 (0.002)	−0.001 (0.001)	0.0005 (0.002)	0.001 (0.001)	0.002 (0.002)	0.003* (0.001)
Education × COVID-19 articles read	0.0003 (0.001)	−0.0001 (0.001)	−0.0004 (0.001)	−0.0003 (0.001)	−0.0005 (0.001)	−0.001* (0.001)
Constant	2.811*** (0.324)	2.867*** (0.376)	3.195*** (0.348)	3.519*** (0.360)	3.332*** (0.360)	2.737*** (0.398)
Observations	465	396	469	399	425	364
*R* ^2^	0.030	0.051	0.026	0.035	0.043	0.050
Adjusted *R*^2^	0.019	0.039	0.016	0.023	0.032	0.037
Residual std. error	1.125 (df = 459)	1.148 (df = 390)	1.219 (df = 463)	1.097 (df = 393)	1.209 (df = 419)	1.168 (df = 358)
*F* statistic	2.808* (df = 5; 459)	4.199*** (df = 5; 390)	2.515* (df = 5; 463)	2.867* (df = 5; 393)	3.801** (df = 5; 419)	3.786** (df = 5; 358)

* *p* < .05. ***p* < .01. ****p* < .001.

**Table A7.** Analysis Split by Country for Table 3 (Model 8).

Predictors and model fit statistics	Dependent variable	
	COVID-19 origin	
	Switzerland	Germany
	1	2
Education	0.369** (0.113)	0.396*** (0.101)
Age	−0.002 (0.004)	0.008 (0.005)
Gender (women)	−0.212 (0.118)	−0.074 (0.124)
COVID-19 origin articles	0.045 (0.029)	0.230*** (0.064)
Education × COVID-19 origin articles	−0.013 (0.011)	−0.083*** (0.024)
Constant	3.318*** (0.354)	2.635*** (0.395)
Observations	425	364
*R* ^2^	0.045	0.067
Adjusted *R*^2^	0.034	0.053
Residual std. error	1.208 (df = 419)	1.158 (df = 358)
*F* statistic	3.941** (df = 5; 419)	5.103*** (df = 5; 358)

** *p* < .01. ****p* < .001.

## Appendix 2: Education Categories in Survey Items

### Switzerland

1. Obligatorische Schule nicht abgeschlossen (Compulsory school not completed)
2. Obligatorische Schule abgeschlossen (Compulsory school completed)
3. 10. Schuljahr, allg. Berufsvorbereitung, 1-jährige Handelsschule, Haushaltslehrjahr oder Sprachaufenthalt (tenth school year, general vocational preparation, 1-year commercial school, household year, or language stay)
4. Anlehre (Apprenticeship)
5. Berufslehre (Vocational training)
6. Vollzeitberufsschule (Full-time vocational school)
7. Fachmittelschule (Specialized middle school)
8. Maturitätsschule, Berufsmaturität, Lehrerseminar, Schule für Unterrichtsberufe (Matura school, vocational baccalaureate, teacher seminar, school for teaching professions)
9. Eidg. Diplom (Meisterdiplom), Eidg. Fachausweis (Federal diploma, Federal certificate of competence)
10. Techniker- oder Fachschule (Technical or specialized school)
11. Höhere Fachschule, HTL, HWV (Higher technical school, HTL, HWV)
12. Universität, ETH, Fachhochschule, Pädagogische Hochschule (University, ETH, University of Applied Sciences, Pedagogical University)

### Germany

1. Keinen Abschluss (No qualification)
2. Noch Schüler (Still a student)
3. Hauptschule, Volksschule bzw. POS 8./9. Klasse (Main school, primary school or POS eighth/ninth grade)
4. Realschule, mittlere Reife, qualifizierter Sekundarabschluss I bzw. POS 10. Klasse (Secondary school, intermediate maturity, qualified secondary education I or POS tenth grade)
5. Berufsschule (Vocational school)
6. Berufsfachschule bzw. Kollegschule (Vocational college or specialized school)
7. Fachhochschulreife (University of applied sciences entrance qualification)
8. Abitur bzw. EOS 12. Klasse (Abitur or EOS twelfth grade)
9. Meisterausbildung (Master craftsman training): 9
10. Fachschule bzw. Fachakademie (Technical school or specialized academy)
11. Universität, Fachhochschule, Pädagogische Hochschule oder Berufsakademie (University, University of Applied Sciences, Pedagogical University or Vocational Academy)

## Notes

1. The source code for the backend (https://gitfront.io/r/user-6760823/Xkff33k5rYKD/webtrack-server/) and frontend (https://gitfront.io/r/user-6760823/FGeoQFp11QgS/webtrack-extension/) implementations of WebTrack is openly available on GitHub.
